# Antibody Quality and Protection from Lethal Ebola Virus Challenge in Nonhuman Primates Immunized with Rabies Virus Based Bivalent Vaccine

**DOI:** 10.1371/journal.ppat.1003389

**Published:** 2013-05-30

**Authors:** Joseph E. Blaney, Andrea Marzi, Mallory Willet, Amy B. Papaneri, Christoph Wirblich, Friederike Feldmann, Michael Holbrook, Peter Jahrling, Heinz Feldmann, Matthias J. Schnell

**Affiliations:** 1 Emerging Viral Pathogens Section, National Institute of Allergy and Infectious Diseases, National Institutes of Health, Bethesda, Maryland, United States of America; 2 Laboratory of Virology, Division of Intramural Research, National Institute of Allergy and Infectious Diseases, National Institutes of Health, Hamilton, Montana, United States of America; 3 Department of Microbiology and Immunology, Jefferson Medical College, Thomas Jefferson University, Philadelphia, Pennsylvania, United States of America; 4 Integrated Research Facility, National Institute of Allergy and Infectious Diseases, National Institutes of Health, Fort Detrick, Maryland, United States of America; 5 Jefferson Vaccine Center, Jefferson Medical College, Thomas Jefferson University, Philadelphia, Pennsylvania, United States of America; US Army Medical Research Institute of Infectious Disease, United States of America

## Abstract

We have previously described the generation of a novel Ebola virus (EBOV) vaccine platform based on (a) replication-competent rabies virus (RABV), (b) replication-deficient RABV, or (c) chemically inactivated RABV expressing EBOV glycoprotein (GP). Mouse studies demonstrated safety, immunogenicity, and protective efficacy of these live or inactivated RABV/EBOV vaccines. Here, we evaluated these vaccines in nonhuman primates. Our results indicate that all three vaccines do induce potent immune responses against both RABV and EBOV, while the protection of immunized animals against EBOV was largely dependent on the quality of humoral immune response against EBOV GP. We also determined if the induced antibodies against EBOV GP differ in their target, affinity, or the isotype. Our results show that IgG1-biased humoral responses as well as high levels of GP-specific antibodies were beneficial for the control of EBOV infection after immunization. These results further support the concept that a successful EBOV vaccine needs to induce strong antibodies against EBOV. We also showed that a dual vaccine against RABV and filoviruses is achievable; therefore addressing concerns for the marketability of this urgently needed vaccine.

## Author Summary

Ebola virus (EBOV) has been associated with outbreaks in human and nonhuman primate populations since 1976. With a fatality rate approaching 90%, EBOV is one of the most lethal infectious diseases in humans. The increased frequency of EBOV outbreaks along with its potential to be used as a bioterrorism agent has dramatically strengthened filovirus vaccine research and development. While there are currently no approved vaccines or post exposure treatments available for human use, several vaccine candidates have shown to protect nonhuman primates from lethal EBOV challenge. Our primary focus is to develop vaccine candidates to protect humans and endangered wildlife species at risk of infection in Africa. Here, we evaluated the efficacy and immunogenicity of our dual vaccines against EBOV and rabies virus (RABV) in rhesus macaques. Our live replication-competent vaccine provided 100% protection following EBOV challenge while the replication-deficient and inactivated candidates provided 50% protection. Interestingly, protection is dependent on the quality of the antibodies rather than the quantity. All three RABV-based EBOV vaccines did induce antibody levels necessary for protection from RABV infection. These results encourage the further development of these novel dual vaccines directed against two of the most lethal viral diseases.

## Introduction

Several members of the *Ebolavirus* genus and *Marburgvirus* genus, Family *Filoviridae*, cause severe and often fatal viral hemorrhagic fever in humans and nonhuman primates [1]. While the public health burden of filovirus infections remains low relative to other public health threats in Africa, outbreaks continue to affect the Central African region including recent outbreaks in Uganda and the Democratic Republic of the Congo in 2012. The high case fatality rate, the increasing public health threat in Africa, and the biodefense concerns associated with these viruses have resulted in considerable activity in filovirus vaccine development [2], [3]. Several vaccination strategies, including DNA, adenovirus, recombinant vesicular stomatitis virus (rVSV), virus-like particles (VLPs) and recombinant parainfluenza virus vectored vaccines, have been developed to deliver primarily the EBOV glycoprotein (GP) as antigen and have been shown to confer protection in animal models [2], [4]–[7]. While each vaccine strategy has shown promising results and is protective in macaques, concerns such as vaccine safety, preexisting vector immunity, manufacturing, or lack of commercial interest have slowed progress.

Recent investigations have focused on the identification of immune parameters that might serve as correlates of protection in vaccinated nonhuman primates (NHPs). The majority of evidence suggests that IgG antibody levels are important for protection in immunized macaques (adenovirus or rVSV-vectored EBOV GP) although the contribution of neutralizing antibodies to protection is unclear [8], [9]. Further support for the potential contribution of antibodies to protection was recently provided by two studies demonstrating that passive transfer of purified IgG from NHP survivor sera or neutralizing monoclonal antibody cocktails could confer protection from Ebola or Marburg virus infections [10], [11]. In addition Marzi *et. al*. showed that the rVSV mechanism of protection for EBOV is mediated by antibodies [12].

EBOV-specific cellular immune responses have also been characterized after several immunization strategies including DNA/adenovirus and VLPs [7]. Using T cell depletion experiments, Sullivan *et al.* recently concluded that EBOV-specific CD8^+^ T cells and not humoral immunity mediated protection from EBOV infection upon adenovirus/EBOV-GP immunization [13]. Collectively, these studies suggest that immune parameters that correlate with and/or confer protection may be multi-factorial and vary by vaccination platform. However, we also need to consider that there are likely different requirements for the induction of anti-EBOV immunity and the recall response after exposure to the pathogen. It is not likely that long-lived immunity can be achieved without T-helper cells. In the case of GP-specific antibodies it needs to be shown that they are maintained over time or CD4^+^ T helper cells will be required to mount fast responses after infection.

A filovirus vaccine would be directed for use in humans at risk of infection in Africa as well as for laboratory workers, healthcare providers, first responders, soldiers, or travelers. Furthermore, EBOV vaccines could be utilized in endangered wildlife species such as gorillas and chimpanzees in Central Africa where they are at risk of lethal EBOV disease. Epidemiologic studies have indicated that EBOV outbreaks have resulted in numerous deaths of these animals in Gabon and the Democratic Republic of Congo, hindering conservation efforts to protect these populations [14]–[16]. A vaccine to protect these at risk NHPs would have a second critical benefit to humans. As EBOV is a zoonotic disease with documented human outbreaks, which can arise from contact with diseased NHPs [17], prevention of disease in these animals might reduce the frequency of EBOV transmission into humans resulting in reduced frequency of outbreaks.

Our goal is to identify a vaccine platform for EBOV and other filoviruses of public health importance that would (a) produce promising candidates for use in both humans and endangered wildlife species and (b) yield multiple vaccine candidates increasing the likelihood that an optimal balance between reactogenicity and immunogenicity might be achieved. To this end, we have utilized the rabies virus (RABV) vaccine platform to develop (a) replication-competent, (b) replication-deficient, and (c) chemically inactivated vaccines expressing EBOV GP (strain Mayinga) [18]. As RABV is still a considerable public health issue in Africa with an estimated 24,000 deaths reported yearly [19]–[21], a bivalent vaccine that confers protection from RABV and EBOV would be an economical and efficient public health tool. The RABV vaccine platform has proven to be an excellent vaccine vector for safe induction of immunity to HIV, SARS-CoV, and hepatitis C virus [22]–[26]. Further attenuated RABV-vectored vaccines have been generated by the deletion of the RABV glycoprotein (G) gene and propagation of viruses on trans-complementing cell lines that express RABV G [25], [27], [28]. Additionally, beta-propiolactone-mediated inactivation of RABV-vectored vaccines has been used to generate killed vaccine candidates against hepatitis C virus and *bacillus anthracis* with optimal safety profiles [22], [29]. Our primary focus is the development of an inactivated vaccine for use in humans based on the potential for superior safety and the successful history of the existing beta propiolactone-inactivated RABV vaccine that is widely used in humans. However, in addition to the development of inactivated RABV/EBOV vaccines, the parental recombinant RABV vaccine used to generate the RABV/EBOV vaccine candidates is derived from the SAD B19 strain which is used for wildlife vaccination by baiting in Europe suggesting additional applications of our vaccine candidates [30], [31]. Therefore, live attenuated RABV/EBOV vaccines could be considered for use in Africa in an analogous campaign to protect at risk NHPs from lethal EBOV infections.

## Results

### Immunogenicity of RABV-based vaccines in NHPs

**Figure 1:**
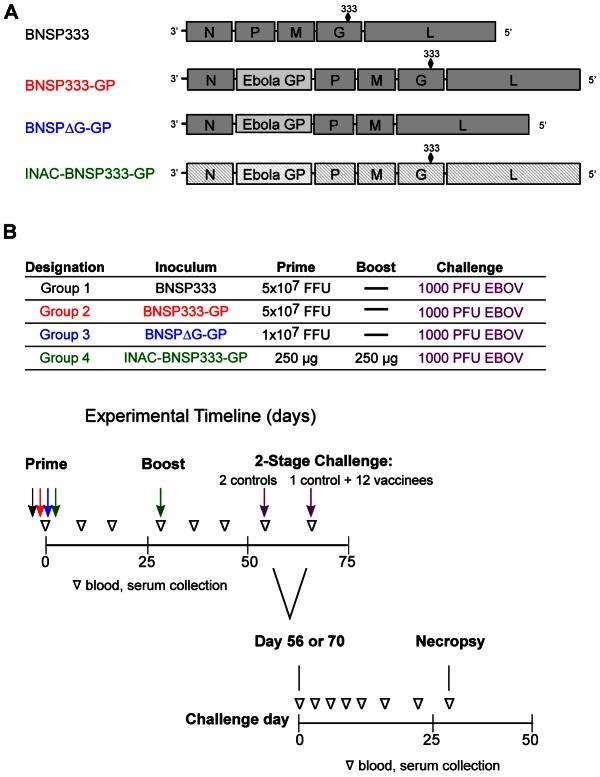
Immunization schedule and RABV/EBOV vaccine constructs. (A) Schematic of the RABV vaccine constructs expressing EBOV GP used for prime and boost immunizations. (B) Experimental timeline: All NHPs were immunized on day 0 and challenged intramuscularly with 1,000 PFU of EBOV on day 56 or 70 as described in the Materials and Methods. The day of challenge (day 56 or 70) is referred to throughout the paper as challenge day 0. Group 4 was boosted with 250 µg of the inactivated construct on day 28.

Our previous research with RABV-based vaccine vectors expressing HIV-1 antigens indicated that such vaccines are highly immunogenic in NHPs against both the RABV-based vector and foreign antigens [25], [32]. However, only replication-competent vaccine vectors expressing HIV-1 GagPol or Env have been analyzed in NHPs, and immunogenicity against filovirus antigens expressed in the RABV vector needs to be evaluated in the NHP model. Here, we analyzed the immunogenicity of three different RABV/EBOV vaccine vectors in NHPs, namely replication-competent (BNSP333-GP), replication-deficient (BNSPΔG-GP) and inactivated virions (INAC-BNSP333-GP) expressing or carrying EBOV GP. The empty, replication-competent vector (BNSP333) served as a control (Figure 1A, [18]).

**Figure 2:**
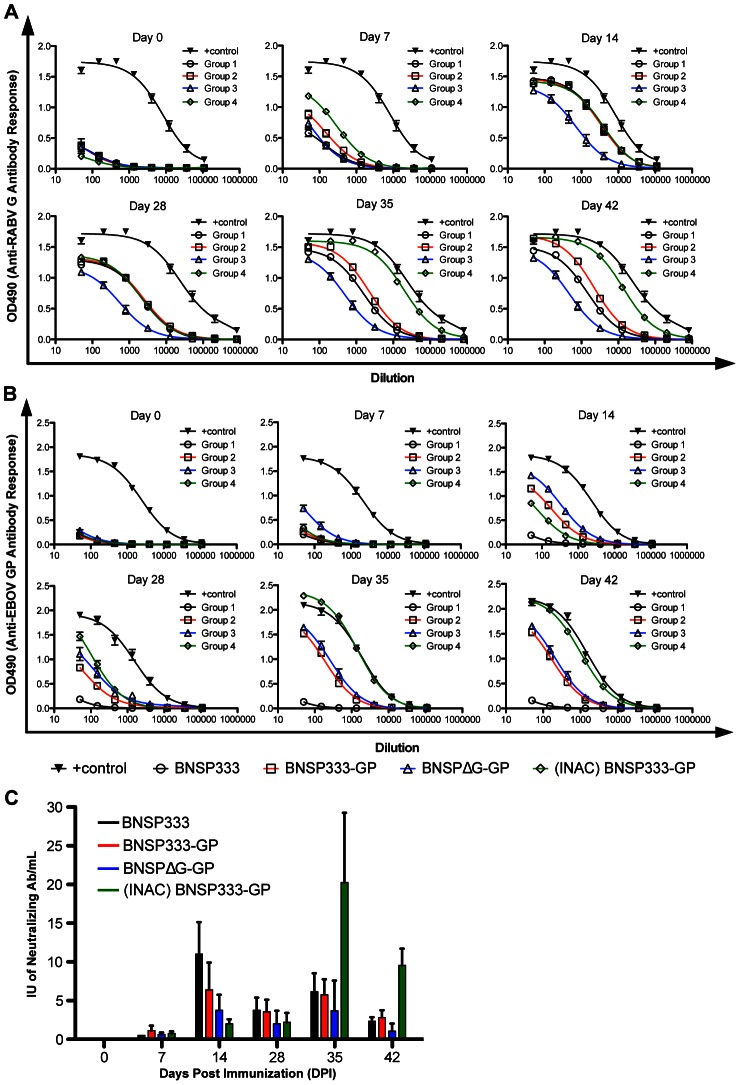
Humoral immune response to RABV G and EBOV GP before Ebola Zaire challenge. (A) Rhesus macaque total IgG immune response to RABV G. OD490 readings were compared to a World Health Organization (WHO) standard (human sera) for rabies. (B) Rhesus macaque total IgG immune response to EBOV GP compared to a control macaque which survived EBOV infection. All sera were diluted 1∶50 and analyzed in a 3 fold serial dilution via ELISA. (C) Neutralization assay for RABV G post immunization.

As outlined in the immunization schedule in Figure 1B, four groups of rhesus macaques were immunized intramuscularly (i.m.) in the caudal thigh muscle as follows: group 1, three NHPs, 5×10^7^ focus-forming units (FFU) BNSP333, black; group 2, four NHPs, 5×10^7^ FFU BNSP333-GP, red; group 3, four NHPs, 1×10^7^ FFU BNSPΔG-GP, blue; group 4, four NHPs, 250 µg purified INAC-BNSP333-GP, green. We followed the immune response of the vaccinated animals over time after vaccination as well as after challenge (Figure 1B). Notably, the goal of this novel vaccine approach was to develop a vaccine that protects from two different highly lethal diseases, rabies and filovirus induced hemorrhagic fever. Therefore, we followed both RABV and EBOV GP-specific immune responses. As shown in Figure 2A, all three vaccines and the empty control vector induced seroconversion against RABV G as early as day 7 after immunization, with increasing IgG levels at day 14 and slightly decreasing levels for the sera collected at day 28. In contrast, at day 7, EBOV GP-specific humoral responses were only detected in sera from animals vaccinated with the replication-deficient vaccine (Figure 2B). On day 14, all groups (groups 2–4) showed a positive signal in the EBOV GP-specific ELISA whereas for the control animals (group 1) only background signals were detected (Figure 2B). Interestingly, the replication-deficient (RABV G-deleted) vector expressing EBOV GP induced the highest EBOV GP-specific responses but the lowest RABV G responses. This is most likely due to the fact that this virus does not encode RABV G and the RABV G-specific immune response results from the G protein contained in the initial vaccine particle preparation [27]. Our previous research on live RABV-based vaccines indicated that pre-existing anti-RABV antibodies prevent a successful secondary immunization; therefore, only the group of rhesus macaques primed with the inactivated RABV virions containing EBOV GP received a boost at day 28 with the same vaccine (Figure 1B). The boost increased the humoral responses against EBOV GP as well as RABV G for group 4 significantly (Figure 2A and B, day 35 and 42). Remarkably, even the animals that were not boosted showed an increase in humoral responses directed against RABV G and EBOV GP from days 28 to 35, indicating that the vaccines were still stimulating the immune system.

High ELISA titers of anti-RABV G antibodies are predictive of protection of the immunized host, but we still wanted to confirm the humoral response against RABV by virus neutralization assays (VNA). The result presented in Figure 2C indicates that all four vaccines induced virus-neutralizing antibodies as early as 7 days after immunization. Notably, the VNA titers were well above the critical level of 0.5 international units (I.U.), which is considered protective from RABV infection in humans [33]. Similar to the total IgG levels against RABV G, we detected an increase of the RABV-specific immune responses by VNA for all vaccine groups. In contrast to RABV, we were unable to detect significant levels of virus-neutralizing antibodies directed against EBOV for groups 2–4 compared to the controls of group 1 (data not shown). We also analyzed the cellular responses utilizing an IFN-γ specific ELISPOT from larger blood samples collected at day 14 and 42. As shown in Table 1, animals of the control group did not mount any cellular responses when stimulated with EBOV GP-specific peptide pools. The highest responses were detected for animals immunized with the replication-competent vaccine, followed by the replication-deficient and the killed viral particles at day 14. However, in each group we failed to detect cellular responses in one or two animals. At day 42, cellular responses were only detected in two animals, which previously had the highest responses. All other animals showed only a background level of EBOV GP-specific cellular responses.

**Table 1:** ELISPOT GP response on frozen cells (spots per million cells).

Group	NHP ID	Sex	Day 14	Day 42	Challenge Day 0	Challenge Day 6
BNSP333	1	F	0.0	0.5	75.0	6.7
	2	M	7.5	0.0	138.3	44.7
	3	F	0.0	0.0	258.3	78.3
BNSP333-GP	4	F	402.5	36.5	963.3	5565.0
	5	M	1112.5	206.0	3261.7	6218.3
	6	F	12.5	2.5	70.0	3668.3
	7	M	95.0	0.5	35.0	5251.7
BNSPΔG-GP	8	F	80.0	1.5	105.0	3.3
	9	M	52.9	1.5	0.0	0.0
	10	M	257.1	5.0	120.0	6551.7
	11	F	165.7	0.5	525.0	6183.3
INAC-BNSP333-GP	12	M	54.3	4.5	248.3	5903.3
	13	M	50.0	0.0	136.7	856.7
	14	F	2.9	0.5	140.0	55.0
	15	F	51.4	2.5	191.7	60.0

### Outcome of challenge with EBOV in vaccinated animals

**Figure 3:**
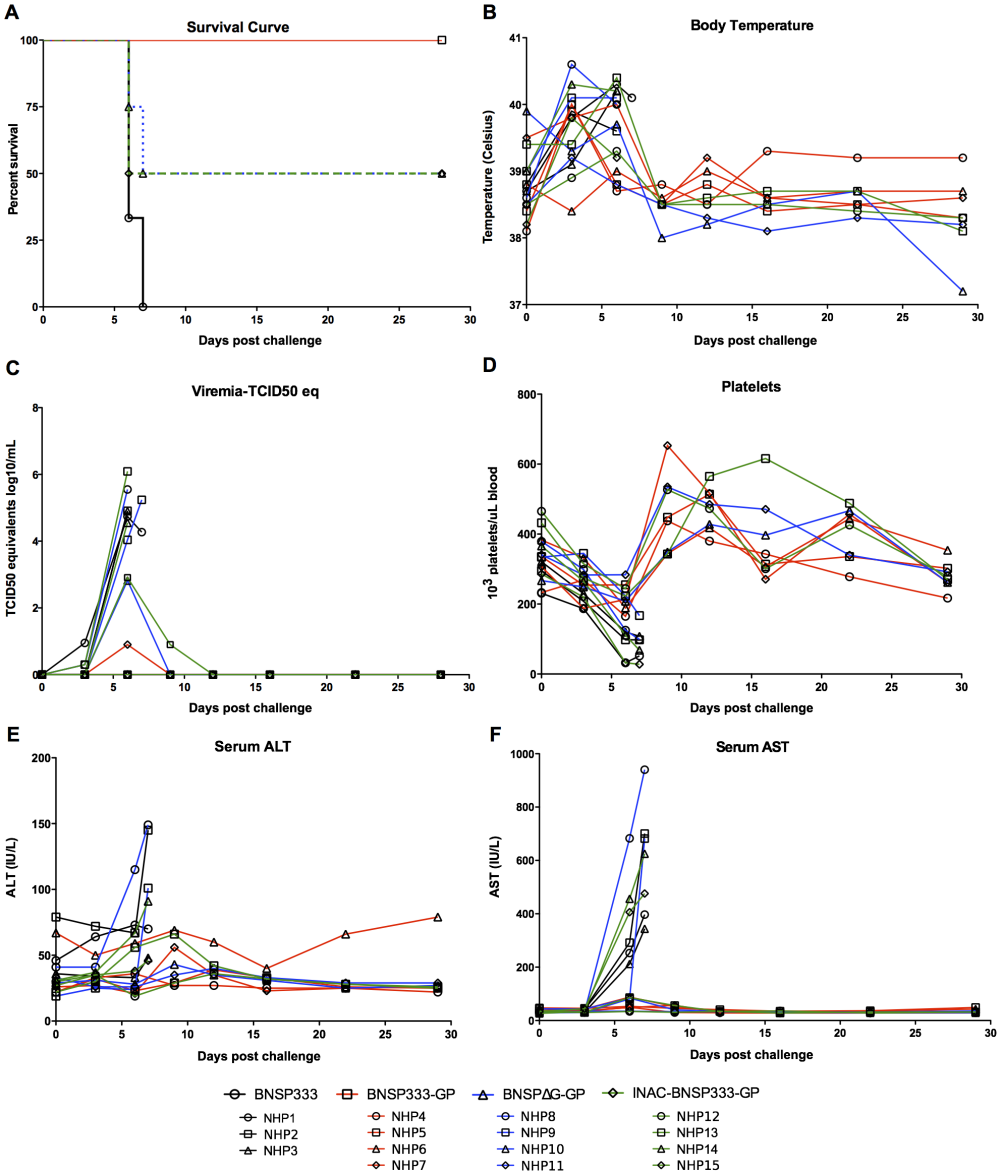
Survival curve and clinical findings in rhesus macaques after EBOV challenge. Rhesus macaques were intramuscularly challenged with 1000 PFU of EBOV on day 56 or 70 (challenge day 0). The Kaplan-Meier survival curve (A) indicates that 100% of animals immunized with the replication-competent vaccine survived EBOV challenge. Clinical signs of infection such as body temperature (B), viremia (C), platelet count (D), serum alanine aminotransferase (ALT) levels (E), and serum aspartate aminotransferase (AST) levels (F) were monitored daily.

After day 42, all animals were transferred to the NIAID BSL-4 facility at the Rocky Mountain Laboratories for EBOV challenge. Since the challenge virus stock had never been utilized in rhesus macaques, we infected two of the three control animals (NHP1 and NHP2) on day 56 with 1000 PFU of EBOV (strain Mayinga) prior to the other animals to ensure the pathogenicity of the virus stock. NHP1 and NHP2 rapidly developed disease and reached the hemorrhagic state (rash) on day 6 and 7 post challenge, respectively, at which point animals had to be euthanized according to approved protocol. Based on this finding, we infected the remaining 13 animals on day 70 with the same challenge virus stock and dose. For each challenge experiment, physical exams and blood draws were performed at day 0, 3, 6, 12, 16, 22, and 28 post-challenge. The outcome of the EBOV challenge and the clinical findings are presented in Figure 3. As show in Figure 3A, all animals immunized with the live replication-competent vaccine, BNSP333-GP (group 2), survived the challenge. As expected, all animals in the control group (NHP 1–3) had to be humanely euthanized according to approved protocol mainly based on high viremia at day 6 (Figure 3C) and rash. Two of four animals from group 3, which were immunized with the replication-deficient vaccine (NHP8 and NHP9), and two of four animals from group 4, which received the inactivated RABV/EBOV particles (NHP14 and NHP15), had to be euthanized. The viral loads detected in the blood of these animals (NHP8, NHP9, NHP14 and NHP15) did not differ from those of the three control animals NHP1-3 (Figure 3C). Interestingly, five out of the twelve animals (NHP4, NHP5, NHP6, NHP11 and NHP12) controlled challenge virus replication with undetectable viremia, whereas for three animals (NHP7, NHP10 and NHP13) the challenge virus was detected transiently at one (group 2 and 3) or two time points (group 4) (Figure 3C). The lack of protection was also reflected clinically (Figure 3B, D–F). Animal body temperature, on average, increased within the first few days after challenge but returned to the original temperatures near day 12 post challenge (Figure 3B). Platelet count significantly decreased after challenge for the unprotected animals whereas protected animals regained normal platelet levels by day 12 post challenge (Figure 3D). Serum alanine aminotransferase (ALT) (Figure 3E) and serum aspartate aminotransferase (AST) levels (Figure 3F) monitored liver function. Elevated levels, as seen by days 3–6, indicated liver damage as a result of EBOV infection.

### Immune responses detected after challenge

**Figure 4:**
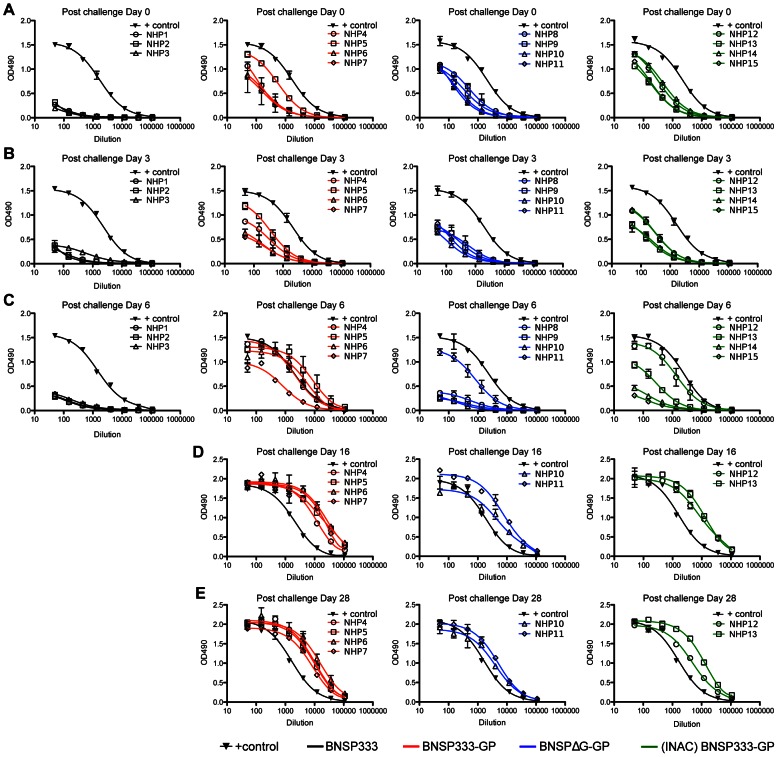
Humoral immune response to EBOV GP after challenge. Rhesus macaque total IgG immune response to EBOV GP for post challenge day 0 (A), day 3 (B), day 6 (C), day 16 (D), and day 28 (E). NHP responses were compared to the response of a control macaque which survived EBOV infection. All sera were diluted 1∶50 and analyzed in a 3 fold serial dilution via ELISA. NHPs belonging to Group 1, those immunized with BNSP333, succumbed to EBOV infection by post challenge Day 9 whereas NHPs in (D) and (E) survived Ebola Zaire challenge.

We also monitored the immune responses of vaccinated animals after challenge during the acute and convalescent phases of disease. As shown in Figure 4, the three animals of the control group failed to mount any EBOV GP-specific responses during the course of the EBOV infection. In contrast, all three groups of vaccinated NHPs (groups 2, 3 and 4) had similar levels of anti-EBOV GP antibodies at the day of challenge (Figure 4A, challenge day 0). The EBOV GP-specific antibodies remained at these levels 3 days after challenge (Figure 4B, challenge day 3). Interestingly, a rapid increase to high levels of EBOV GP-specific IgG was observed on day 6 after challenge in the serum of the five animals that survived (NHP4, NHP5, NHP6, NHP11, and NHP12). Notably, these high antibody levels were detected in those animals that did not have detectable levels of EBOV RNA in their blood during the course of challenge. The three animals (NHP7, NHP10, and NHP13), which did show a lower but significant increase in EBOV GP-specific antibodies 6 days after challenge, demonstrated transient levels of EBOV viremia but survived (figure 3C). Lastly, we failed to detect an increase in the humoral responses against EBOV GP in the serum of four animals on day 6 post challenge (NHP8, NHP9, NHP14, and NHP15). These were the animals that had to be humanely euthanized. Based on these results, we concluded that one requirement for a successful RABV-based EBOV vaccine is the rapid recall response of humoral immunity against EBOV GP after EBOV challenge. The results presented above also indicate that virus specific antibodies are important to control EBOV infection. However, the antibody titers against EBOV GP at the day of challenge were similar for all three groups and, more importantly, the same within groups 3 and 4 where two animals in each group were protected while the other two were not. Therefore, we decided to analyze the humoral immune responses in greater detail.

**Figure 5:**
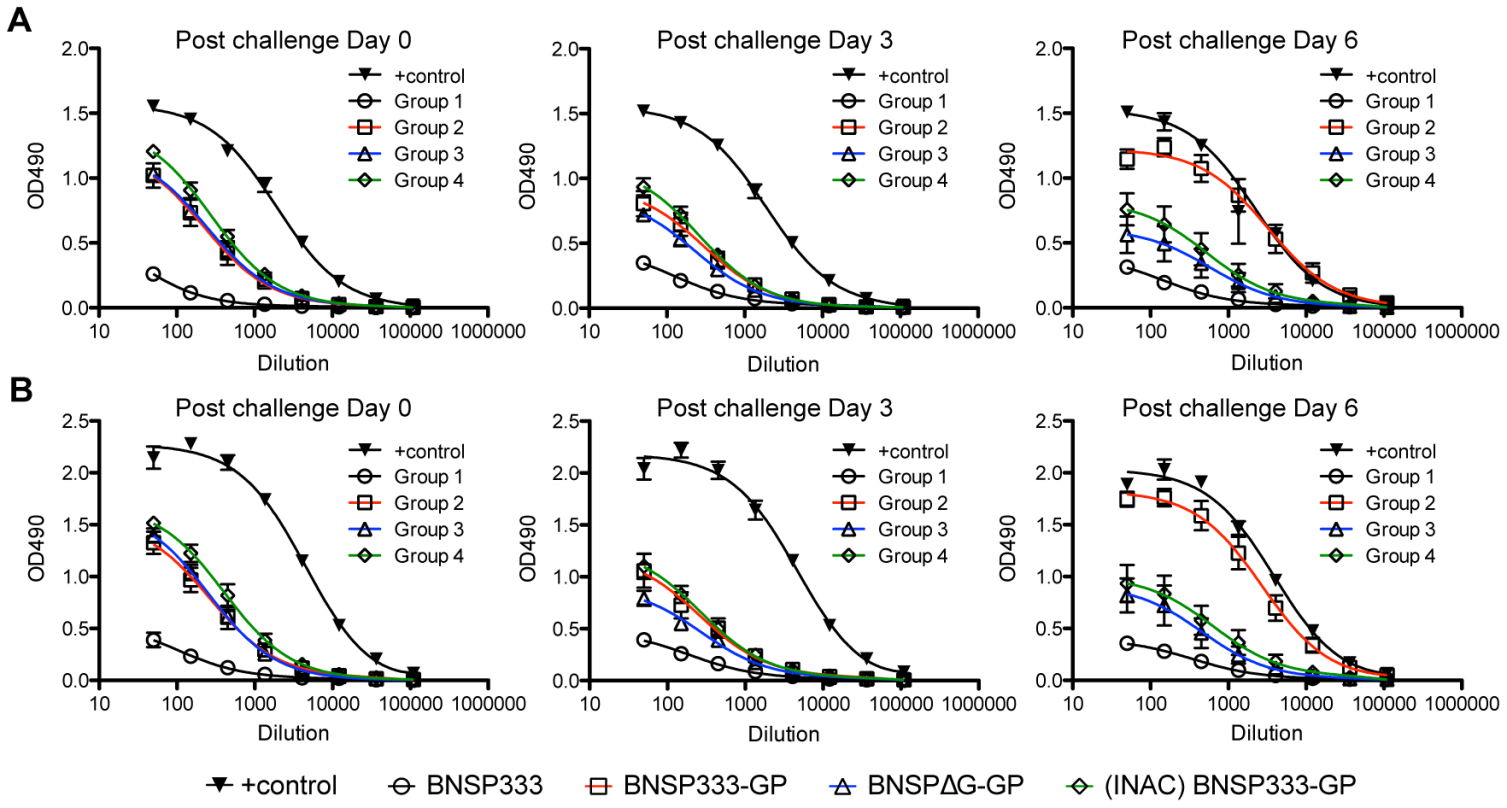
Full length EBOV GP and EBOV GP-ΔMLD exhibit similar immunogenicity. Total IgG response to full length EBOV GP (A) and EBOV GP-ΔMLD (B) on days 0, 3, and 6 post challenge.

First, we performed an EBOV GP specific ELISA utilizing full-length EBOV GP as well as a “mucin-like domain” (MLD) deleted version (EBOV GP-ΔMLD). The MLD is a heavily glycosylated region of the EBOV GP ectodomain. Previous research indicates that antibodies directed against the MLD not only fail to neutralize EBOV but can even enhance the infection [34]. Results shown in Figure 5 demonstrate that similar humoral responses were detected for sera from all animals utilizing both full-length EBOV GP (Figure 5A) and EBOV GP-ΔMLD (Figure 5B) ELISAs. We concluded from these findings that there are no significant differences in the target of the induced antibodies for these three vaccines, at least not in regard to the MLD.

**Figure 6:**
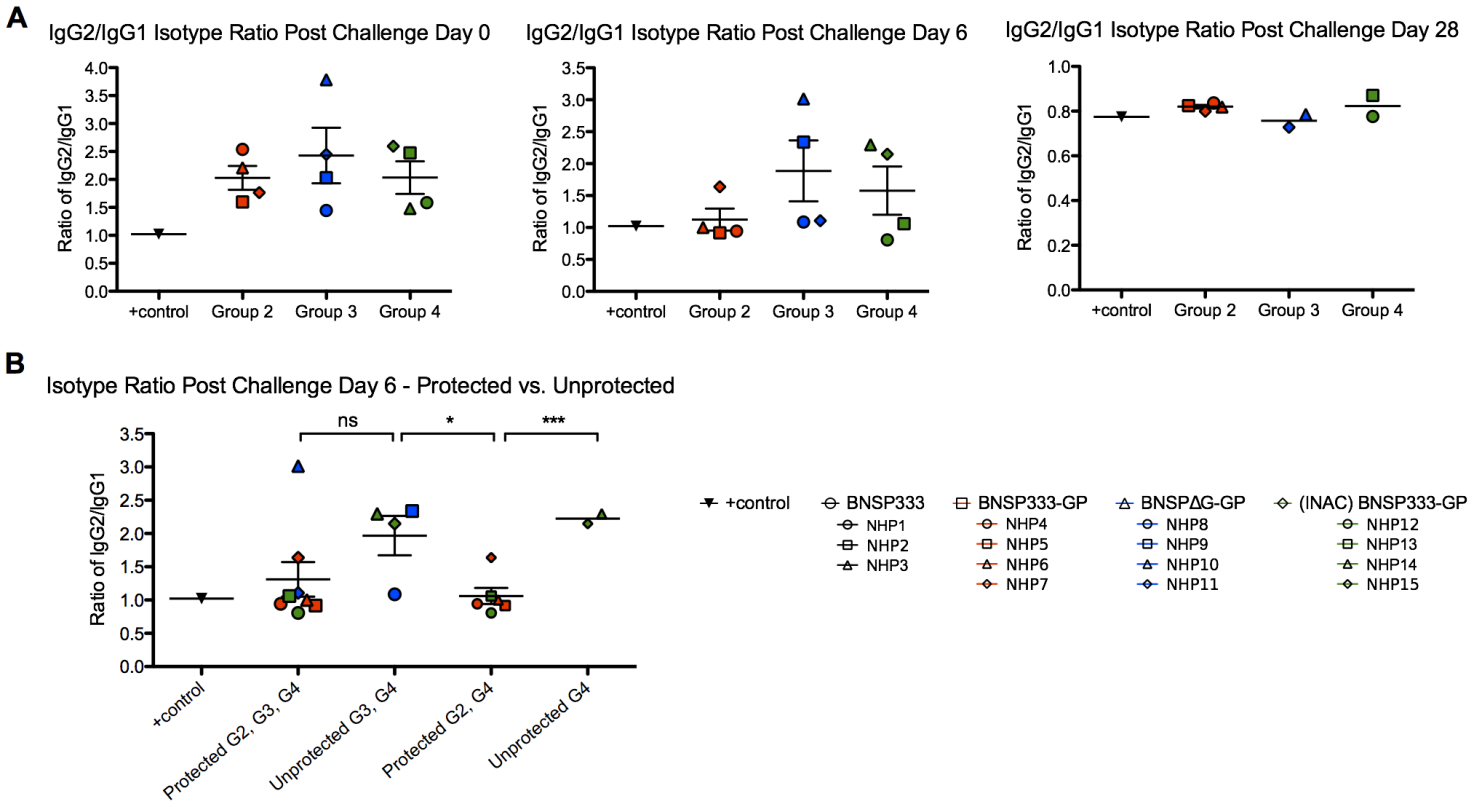
IgG2/IgG1 isotype ratios in response to EBOV GP. (A) Isotype ratios (IgG2/IgG1) at 1∶150 for post challenge days 0, 6, and 28. Ratios less than 1.0 indicate a bias towards a Th1 response. Group 1 animals did not show an IgG1 or IgG2 response to EBOV GP so the ratios are not shown. (B) Isotype ratios for protected animals versus unprotected animals after EBOV challenge. The isotype ratios of the protected animals were not statistically significant when compared to the ratios of the unprotected animals. When analyzing the isotype ratios of group 2 and group 4 protected animals to the unprotected animals in group 4, there is a significant difference (***, p<0.001). Statistical analysis was performed using unpaired *t*-test with Welch's correction to compare two groups. Results shown are presented as the mean. *p<0.05, **p<0.01, ***p<0.001.

As there seem to be no obvious differences in the EBOV GP-target of the antibodies within the different groups of vaccines, we analyzed qualitative differences of the antibodies. Th1 dependent IgG1 antibodies in NHPs have been shown to possess better antiviral properties by mediating antibody-dependent cellular cytotoxicity and complement activation [35]. Therefore, we performed an isotype-specific ELISA utilizing full-length EBOV GP to determine if any differences in the antibody isotypes occurred between the vaccines. As shown in Figure 6, the positive control NHP ratio of IgG2/IgG1 on day 0 post-challenge was around 1.0, whereas this ratio was an average of about 2.0 for all the vaccinated animals from groups 2–4. However, six days after challenge this IgG2/IgG1 ratio changed to ∼1.0 for the sera collected for the animals from group 2, which is the group where all four animals survived. In contrast, the average of the IgG2/IgG1 ratio for group 3 was 2.0 and for group 4 was 1.5. In both of these groups two animals were not protected. These data suggest that IgG1-biased humoral responses might be beneficial for the control of EBOV infection. This contention was further supported by the analysis of the individual animals in groups 2 and 4. As shown in Figures 3 and 6, NHP7 (group 2) had detectable EBOV RNA in the blood on day 6 and also had the highest IgG2/IgG1 ratio of 1.6. The same is true for group 4 where both protected animals had an IgG2/IgG1 ratio below 1.0, whereas the unprotected animals were above 2.0. The only exception from this observation is one protected animal in group 3 (NHP10), which had a clear bias towards an IgG2 response indicated by an IgG2/IgG1 of almost 3.0. However, this animal had a very low antibody response even on day 6 after challenge (Figure 4). Lastly, the final outcome at four weeks after challenge was similar for all surviving animals with an IgG1-biased response and an IgG2/IgG1 ratio of about 0.8 (similar to the positive control). In general, this novel finding strongly indicates that an IgG1-biased immune response against EBOV GP is advantageous for protection and should be further evaluated with larger animal numbers and for other vaccine approaches.

**Figure 7:**
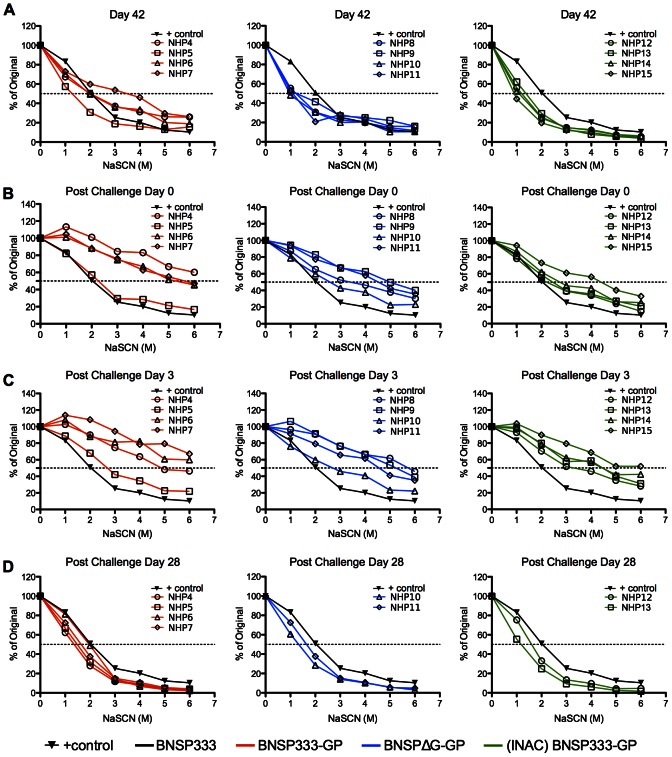
Avidity assay of total IgG immune response to EBOV GP. Sera were analyzed from day 42 (A), day 0 (challenge) (B), day 3 post challenge (C), and day 28 post challenge (D) (study termination) with a NaSCN-displacement ELISA. Serum samples were diluted to an OD490 reading of 0.8 based on total IgG ELISA data.

Lastly, qualitative antibody differences were also analyzed by measuring the avidity of the antibodies before, during, and after challenge in the vaccinated animals of groups 2–4. As shown in figure 7 on day 42, the avidity of the antibodies in the serum of the vaccinated animals was similar (group 2) or below that of the control animals (group 3 and group 4). However, the avidity of EBOV GP-specific antibodies significantly increased until challenge day 0 and was above the level of the control animals for most of the vaccines, indicating that RABV-vector induced immune responses mature over a long period of time. However, we did not find a direct correlation between avidity of the EBOV GP-specific antibody induced by the vaccines and protection from disease.

Two additional interesting findings are worth mentioning. As listed above, it seems that the avidity of the EBOV GP-specific immune response is not complete until after day 42, since the levels continue to increase at least until challenge day 0 (also referred to as day 70), which is the last time point analyzed before the challenge with EBOV (strain Mayinga). Secondly, and even more interestingly, challenge with EBOV does increase the avidity of the antibodies directed to EBOV GP (see Figure 7, challenge day 0 and post challenge day 3). In order to investigate if this is an antigen-specific effect, we analyzed the avidity of the RABV G-specific antibodies and observed a similar increase in avidity of the RABV G-specific antibodies compared to EBOV GP-specific antibodies, which was also confirmed by VNA (supplemental Figure S1). The VNA showed a significant increase in the surviving animals of about 3 to 4-fold. Even though we can only speculate about the mechanism of protection, there is a clear increase of the immune response even against an antigen (e.g. RABV G) not expressed during the challenge virus infection.

## Discussion

We have previously described the generation, propagation, safety, immunogenicity, and protective efficacy of RABV/EBOV vaccine candidates [18], [36], [37]. Two live vaccine candidates, BNSP333-GP and BNSPΔG-GP with a deletion of the entire RABV G gene, were found to be avirulent upon peripheral administration in mice. Based on the efficient incorporation of EBOV GP into the virion, an inactivated vaccine (INAC-BNSP333-GP) was also produced by treatment of the RABV/EBOV vaccine with beta-propiolactone, the standard method utilized for the current human RABV vaccine. Each bivalent vaccine candidate induced strong humoral immunity to RABV G and EBOV GP, and conferred protection from both lethal RABV and mouse-adapted EBOV challenge in mice.

Based on the demonstration of promising safety, immunogenicity, and protective efficacy of the live and inactivated RABV/EBOV vaccines in mice, we sought to evaluate these vaccines in nonhuman primates. All three vaccines did not induce any clinical sings including fever or weight loss after vaccination and we were not able to detect any of the vaccine vectors in the blood of the vaccinated NHP by RT-PCR (data not shown). However, further safety studies are necessary for the replication-competent vector to analyze any impact of EBOV GP for this vaccine. Immunogenicity was examined following challenge with EBOV (strain Mayinga). Each vaccine candidate was found to induce potent humoral immunity and 50% to 100% protection from lethal challenge.

Our results indicate that the protection of immunized animals was largely dependent on the induced humoral immune response against EBOV GP. This finding is not too surprising because acute viral infections are often controlled by antibodies rather than cytotoxic T-cells, which in general are more important for the control of chronic infections [38]. However, previous research did suggest CD8^+^ T-cells as the major player for protection from EBOV infection in a single vaccination strategy [13]. This has been challenged by recent studies indicating that, in general, protection of NHPs by different EBOV vaccines seems to depend on the presence of anti-EBOV GP antibodies as well as EBOV GP-specific CD4^+^ T-helper cells [9], [12]. Moreover, it cannot be excluded that CD8^+^ T-cells play a role in viral clearance but no CD8^+^ memory T-cells are needed. Lastly, the relatively high background in our ELISPOT assay might have prevented us to detect low cellular responses. In any case, our results indicated a major role of EBOV GP-specific antibodies to control the challenge virus replication, and we therefore focused on these responses in greater detail. In this regard, the finding that only 50% of NHPs in groups 3 and 4 were protected was an advantage, because all three groups of vaccinated NHPs did have similar levels of anti-EBOV GP antibodies as analyzed using an EBOV GP-specific ELISA. This suggested a qualitative difference in the humoral responses for the three vaccines.

First we investigated if the anti-EBOV GP antibodies were directed against different regions within the glycoprotein. Previous work by others indicates that antibodies directed against the MLD within EBOV GP can enhance the infection with EBOV [34]. Therefore, we analyzed the anti-EBOV GP humoral responses from all three vaccines utilizing full-length EBOV GP and EBOV GP-ΔMLD. However, there was no significant difference in the ELISA signal for each serum sample to the two versions of EBOV GP, and we concluded that MLD-directed antibodies do not explain the difference in the observed protection.

Secondly, we analyzed if we could detect differences in the avidity of the antibodies for the three groups of vaccinees, but such differences were not detected. Surprisingly, we found that the avidity of the anti-EBOV GP-specific antibodies greatly increased during challenge at a similar percentage for all tested sera. Whereas we cannot explain this increase in antibody avidity, it was transient and not specific to antibodies against EBOV GP, as the avidity of RABV G-specific antibodies also increased.

It is well established that EBOV GP-specific antibodies induced by different EBOV vaccine candidates may have no or only weak VNA activity, but are still protective [8], [9]. These findings indicate that antibody-dependent cell-mediated cytotoxicity (ADCC) might play a major role in protection from EBOV infection. Because ADCC depends on IgG1 antibody responses, we analyzed the total IgG response and also the IgG subtype responses (e.g. IgG1 and IgG2). Our results indicated that all protected animals, independent of the group, had an IgG2/IgG1 ratio of ∼0.8 whereas the unprotected animals had a higher ratio of ∼2.5. Interestingly, two animals from group 3 and group 4, NHP10 and NHP13, transiently had the highest viral loads and also had IgG2/IgG1 ratios that fell between those of unprotected and protected animals. These results clearly indicate that the quality of the antibodies in regard to the isotypes is very important for a successful EBOV vaccine based on EBOV GP. However, the protected animals from group 2 and 4 had also the highest total antibody levels and therefore we conclude that in the case of a rabies-based EBOV vaccine, high level of GP-specific antibodies that are IgG1-bias are very likely to be significant as shown for groups 2 and 4 in Figure 6B.

In summary, the results presented in this study clearly indicate that the RABV-based vector induced an immune response sufficient to protect from lethal EBOV infection. In the case of replication-competent RABV vectors expressing EBOV GP, no further improvements are necessary and such a vector could be used to protect NHPs from EBOV in the endemic setting. Of note, it would be best to establish efficacy via an oral application, which is already well established for live RABV in wildlife [30], [31]. The replication-deficient and the inactivated RABV particles did not protect all animals; therefore, the responses induced by these vaccines need to be improved to resemble the responses detected for the replication-competent vaccine, BNSP333-GP, which did protect 100% of the animals. In any case, for the replication-deficient vector, the virus could be concentrated so higher titers such as the once used for the replication competent vaccine can be used for the immunization. We cannot exclude the possibility that using a five-fold lower titer for the immunizations than was used for BNSP333-GP was responsible for this difference in protection.

Moreover, another vector choice could be the matrix protein (M)-deleted replication-deficient RABV vector expressing EBOV GP. Studies with such a vector as a RABV vaccine indicated that it is superior, even compared to a replication-competent RABV [39]. In the case of the inactivated virions containing EBOV GP, a new construct containing an exact fusion of the RABV G cytoplasmic domain to EBOV GP increased the incorporation level about two-fold (the previous construct contained two foreign amino acids between the EBOV GP transmembrane domain and the RABV G cytoplasmic domain) and showed better responses than the current construct in mice (Willet and Schnell, unpublished data). Moreover, we discovered that the glycosylation pattern of EBOV GP was different for RABV particles grown on BSR cells than particles grown on VERO cells (data not shown). Therefore, we believe that the utilization of particles containing higher levels of EBOV GP and perhaps an additional immunization dose would bring the protection rate to 100% of the animals, a reachable goal for a safe and promising dual vaccine.

## Materials and Methods

### Animal ethics statement

This study was carried out in strict accordance with the recommendations described in the Guide for the Care and Use of Laboratory Animals of the National Institute of Health, the Office of Animal Welfare and the United States Department of Agriculture. All animal work was approved by the NIAID Division of Intramural Research Animal Care and Use Committees (IACUC), in Bethesda, MD (protocol # OSD-28) and at the Rocky Mountain Laboratories (RML, protocol # 2012-004-E). Both facilities are accredited by the American Association for Accreditation of Laboratory Animal Care. All procedures were carried out under Ketamine anesthesia by trained personnel under the supervision of veterinary staff and all efforts were made to ameliorate the welfare and to minimize animal suffering in accordance with the “Weatherall report for the use of non-human primates” recommendations. Animals were housed in adjoining individual primate cages allowing social interactions, under controlled conditions of humidity, temperature and light (12-hour light/12-hour dark cycles). Food and water were available *ad libitum*. Animals were monitored twice daily (pre- and post-challenge) and fed commercial monkey chow, treats and fruit twice daily by trained personnel. Early endpoint criteria, as specified by the RML IACUC approved score parameters, were used to determine when animals should be humanely euthanized.

### NHP immunization and challenge

Fifteen RABV and EBOV seronegative rhesus macaques were assigned to four groups to evaluate the recently developed RABV/EBOV vaccine candidates (Figure 1). Groups of four animals were used for the vaccine candidate groups while a group of three animals served as the negative control. On day 0, group 1 (control) animals were immunized intramuscularly (i.m.) in the caudal thigh with a 5×10^7^ FFU dose of live parent RABV vaccine, BNSP333. Group 2 animals were immunized i.m. with a 5×10^7^ FFU dose of the full length parent RABV vaccine expressing EBOV GP (designated as BNSP333-GP). Group 3 animals were immunized i.m. with a 1×10^7^ dose of the parent vaccine expressing EBOV GP but containing a deletion in the rabies glycoprotein gene (designated BNSPΔG-GP). Group 4 animals were immunized i.m. with 250 µg of beta-propiolactone inactivated BNSP333-GP (designated INAC-BNSP333-GP). Group 4 was boosted with 250 µg inactivated virus on day 28. All macaques were bled on days 0, 1, 3, 5, 7, 14, 28, 35, and 42 before transport from the National Institutes of Health Animal Center (Poolesville, MD) to the National Institutes of Health, Rocky Mountain Laboratories (Hamilton, MT). Since the challenge virus stock had never been utilized in rhesus macaques, we infected two of the three control animals (NHP1 and NHP2) on day 56 with 1000 PFU of EBOV (strain Mayinga). Based on finding that the utilized virus was virulent and caused EBOV hemorrhagic disease, we infected the remaining 13 animals on day 70 with the same challenge virus stock and dose. For each challenge experiment, physical exams and blood draws were performed on day 0, 3, 6, 9, 12, 16, 22, and 28 post-challenge. Serum aliquots treated by gamma-irradiation as per approved protocol were sent to Thomas Jefferson University for analysis by enzyme-linked immunosorbent assay (ELISA).

### Hematology and serum chemistries

The total white blood cell count, lymphocyte, platelet, reticulocyte and red blood cell counts, hemoglobin, hematocrit values, mean cell volume, mean corpuscular volume, and mean corpuscular hemoglobin concentrations were determined from EDTA blood with the HemaVet 950FS+ laser-based hematology analyzer (Drew Scientific, Waterbury, CT). Serum biochemistry was analyzed using the Piccolo Xpress Chemistry Analyzer and Piccolo General Chemistry 13 Panel discs (Abaxis, Union City, CA).

### Viral load

Levels of viral RNA were determined using quantitative RT-PCR (qRT-PCR) as described previously [12]. For determination of virus titers in NHP blood and tissue samples, Vero E6 cells were seeded in 48-well plates the day before titration. Blood samples were thawed and serial dilutions were prepared. Tissues were homogenized in 1 ml plain DMEM and, as with the blood, serial dilutions were prepared. Media was removed from cells and triplicates were inoculated with each dilution. After one hour, DMEM supplemented with 2% FBS, penicillin/streptomycin and L-glutamine was added and incubated at 37°C. Cells were monitored for cytopathic effect (CPE) and 50% tissue culture infectious dose (TCID_50_) was calculated for each sample employing the Reed and Muench method.

### ELISPOT

To evaluate T- cell responses to EBOV GP, NHP PBMCs were tested using the NHP IFNγ ELISPOT Kit (R&D Systems, Cat#EL961) as per the manufacturer's instructions. Briefly, microplates were filled with 200 µl per well of sterile culture media (RPMI-1640, 10% FBS, 1% Penstrep) as blocking media and incubated at room temperature while NHP PBMCs and stimulating antigens were prepared for plating. Antigens were prepared in sterile culture media to achieve final concentrations as follows: GP peptide pool (JPT) at 10 µg/ml; Pokeweed Mitogen (positive control) at 1 µg/ml; Influenza NP peptide (Mimotopes, negative control) at 10 µg/ml. Unstimulated cells were used to normalize spot counts to background levels. Blocking media was removed, and antigen was added respectively. PBMCs were added to respective wells at 1×10^5^ cells/well. Plates were incubated at 37°C, 5% CO_2_ for 48 h. Cells were then removed, and plates were washed four times with Wash Buffer (R&D Systems). Plates were stained and developed according to R&D Systems protocol with Detection Antibody, Streptavidin-AP and BCIP/NBT chromogen. Plates were rinsed with deionized water and allowed to dry completely before scanning and counting using a CTL Immunospot Reader.

### RABV VNA

A modified rapid fluorescent focus inhibition test (RFFIT) was performed to determine RABV neutralizing antibody levels in the immunized NHP sera. Three-fold serial dilutions of sera or WHO standard RABV IgG in Cellgro Complete serum free media (Mediatech) were incubated at 37C for 1 h with BNSP (parent RABV of BNSP-333 that does not have the attenuating mutation at position 333) at a concentration to achieve an moi of 1 at 24 h post-infection in the negative control. Then, the mixture was added to one day old BSR cells (BHK-21 derived cell line) that had been grown in DMEM (Mediatech) supplemented with 10% FBS (Atlanta Biologicals) and 1% penicillin/streptomycin (Mediatech) on 96 well plates, and plates were incubated for 24 h at 34C. Plates were then fixed with 80% acetone and stained with anti-RV N (Fujirebio). Plates were read for percent infected cells per well, and IUs of antibody were calculated based on the WHO standard, where 50% infection accounts for 2 IU.

### EBOV VNA

Neutralizing antibody titers were determined by performing focus reduction neutralization titration assays (FRNT) as described previously [12]. Briefly, Vero E6 cells were seeded into 96 well plates to generate a confluent monolayer on the day of infection. Serum dilutions were prepared in plain DMEM and 25 µl were incubated with 200 ffu EBOV expressing green fluorescence protein (EBOV-GFP) in a total volume of 50 µl. After 60 min at 37°C the media was removed from cells, the serum-virus mixture was added and samples were incubated for 60 min at 37°C. Then the mixture was removed from the cells and 100 µl of 1.2% carboxymethyl cellulose in MEM (2% FBS) was added per well and left for 4 days at 37°C. The neutralizing antibody titer of a serum sample was considered positive at a dilution showing a>80% reduction (FRNT 80) in GFP-foci compared to the control without serum.

### Production of HA-tagged EBOV GP

Sub-confluent T175 flasks of 293T cells (human kidney cell line) were transfected with a hemagglutinin (HA) tagged EBOV GP expression plasmid encoding amino acids 33–632 of the EBOV GP ectodomain (EBOV GP-ΔTM) or a truncated version that lacks amino acids 312–462 of the EBOV GP mucin-like domain (EBOV GP-ΔMLD-ΔTM). Both plasmids were kindly provided by Erica O. Saphire of the Scripps Research Institute, La Jolla, CA. Supernatant was added to an equilibrated anti-HA agarose (Pierce) column containing a 2.5 mL agarose bed volume. The column was washed with 10 bed volumes of TBST (TBS containing 0.05% Tween 20) and 2 bed volumes of TBS before adding 5 mL of 200 µg/mL HA peptide in TBS (Pierce HA peptide). The peptide was added at a flow rate of 500 µL/min and incubated overnight at 4°C. Bound EBOV GP was eluted with 3 mL of 200 µg/mL HA peptide in TBS. Fractions were collected and analyzed for EBOV GP via Western blot with a nitrocellulose membrane and monoclonal anti-HA antibody (Sigma) prepared in 5% BSA/TBST and goat anti-mouse IgG-HRP. EBOV GP positive fractions were dialyzed with 10K MWCO dialysis cassettes (Thermo Scientific) to remove excess HA peptide used to elute the HA-tagged EBOV protein.

### Total IgG and isotype ELISAs specific for EBOV GP and RABV G

Rhesus macaque sera obtained from the NIH were tested to analyze the humoral response to EBOV GP and RABV G. EBOV GP antigen for coating plates was obtained by harvesting supernatant from transfected 293T cells and purifying the secreted protein with an anti-HA agarose column as described above. 96-well plates (Nunc, Immulon 4 HBX) were coated overnight at 4°C with 50 ng/well purified EBOV GP or 100 ng/well purified RABV G in Na_2_CO_3_ coating buffer. Plates were washed three times with PBST (PBS with 0.025% Tween 20) and blocked at room temperature for 1–2 hours with 5% dry non-fat milk in PBST. Serum samples were diluted 1∶50 in 0.5% BSA-PBST and 100 uL was added to each well in a 1∶3 serial dilution. Plates were incubated overnight at 4°C, washed three times with PBST, and incubated for 2 hours with 100 uL/well of goat anti-human IgG-HRP. Plates were washed with PBST and developed with 200 uL/well of SigmaFast o-phenylenediamine dihydrochloride (OPD) substrate. After incubating for 5 minutes at room temperature, the reaction was stopped with 50 uL of 3 M H_2_SO_4_ and the absorbance was read at 490 nm. IgG subclass specific ELISAs were performed for EBOV GP and RABV G with anti-human (Abcam) and anti-rhesus (NIH NHP Reagent Source) antibodies. Plates were incubated with OPD substrate for 8–13 minutes before stopping the reaction with 3 M H_2_SO_4_.

### Antibody avidity assays

Macaque sera were measured for total IgG avidity to RABV G and EBOV GP using a sodium thiocyanate (NaSCN) displacement ELISA to determine the concentration of NaSCN needed to dissociate 50% of the antibody-antigen interactions. The avidity assays were set up similar to the ELISA protocol described above however the sera samples were diluted to the concentration that would yield an OD reading of 0.8 nm. Prior to incubation with the secondary antibody, the plates were treated with increasing concentrations of NaSCN in PBS (0 M, 1 M, 2 M, 3 M, 4 M, 5 M, 6 M) for 15 minutes at room temperature. Wells receiving 0 M NaSCN were incubated with PBS. The plates were immediately washed three times with PBST (0.025% Tween in PBS) before continuing with the ELISA protocol. All avidity assays were performed in triplicate.

### Statistical analysis

All data were analyzed by Prism software (GraphPad, version 5.0 d). Statistical analysis was performed using unpaired *t*-test with Welch's correction to compare two groups and represented as two-tailed p-value with a confidence interval of 95%. Presented results show the mean of measurements within a group. For all statistics, the following notations are used to indicate significance between two groups: *p<0.05, **p<0.01, ***p<0.001.

## Supporting Information

Figure S1 **Avidity of IgG antibodies in response to RABV G and RABV G neutralization assay.** Sera were analyzed on day 28 (A) (INAC BNSP333-GP animals boosted), day 35 (B), day 42 (C), post challenge day 0 (D), day 16 (E), and day 28 (F) with a NaSCN-displacement ELISA. Serum samples were diluted to an OD490 reading of 0.8 nm based on total IgG ELISA data. (G) Neutralization assay for RABV G post challenge.
(TIF)
