# Supplementary figures and images for: Antibody Quality and Protection from Lethal Ebola Virus Challenge in Nonhuman Primates Immunized with Rabies Virus Based Bivalent Vaccine
Source: PLoS Pathog. 2013 May 30;9(5):e1003389. doi: 10.1371/journal.ppat.1003389 (PMC3667758; doi:10.1371/journal.ppat.1003389)

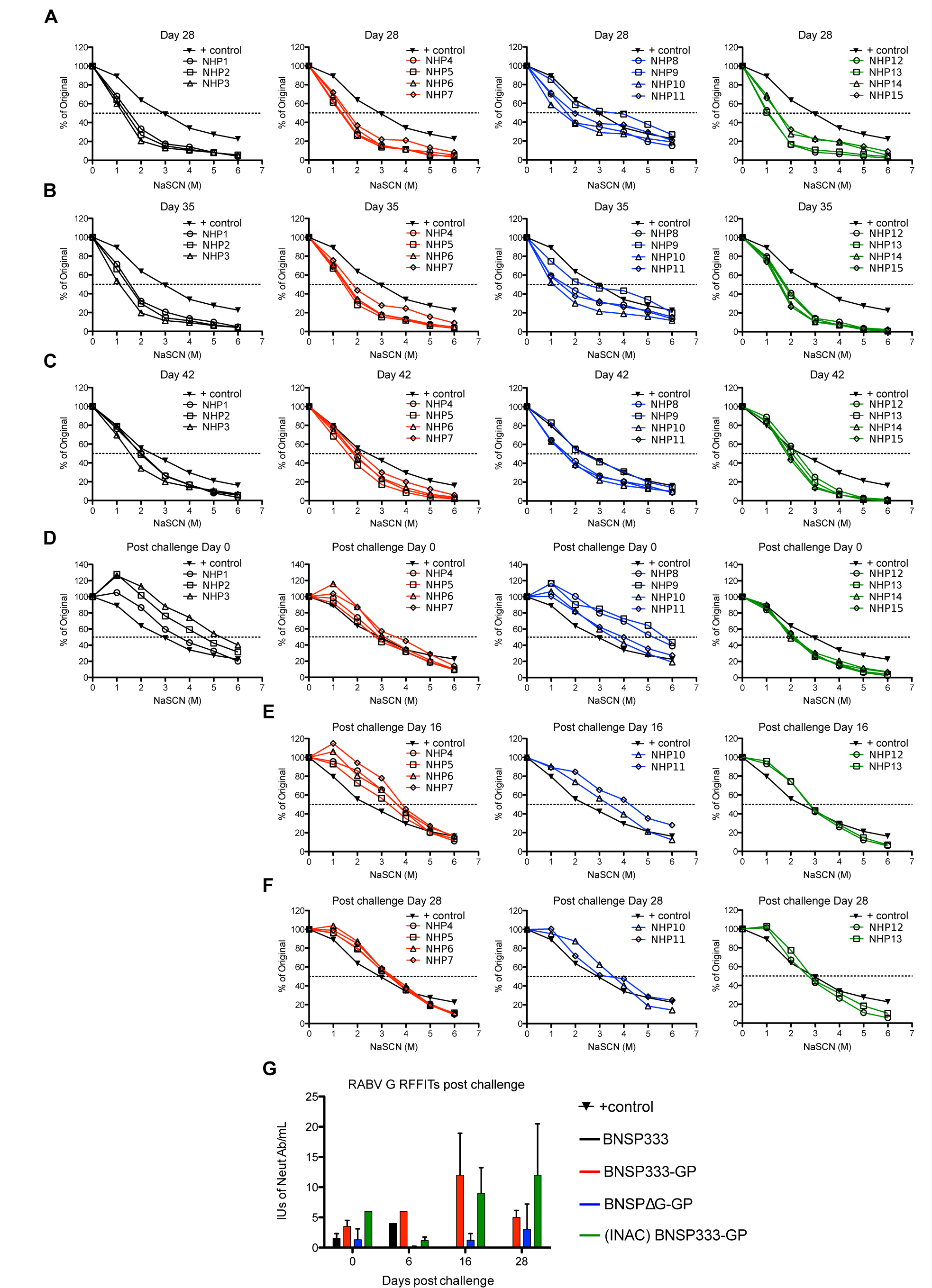

Supplement: Figure S1 — Avidity of IgG antibodies in response to RABV G and RABV G neutralization assay. Sera were analyzed on day 28 (A) (INAC BNSP333-GP animals boosted), day 35 (B), day 42 (C), post challenge day 0 (D), day 16 (E), and day 28 (F) with a NaSCN-displacement ELISA. Serum samples were diluted to an OD490 reading of 0.8 nm based on total IgG ELISA data. (G) Neutralization assay for RABV G post challenge. (TIF) [file ppat.1003389.s001.tif]
